# The Rapid CarbaLux Combination Test to Uncover Bacterial Resistance and Heteroresistance Prior to Antibiotic Treatment

**DOI:** 10.3390/diagnostics15202624

**Published:** 2025-10-17

**Authors:** Hans Rudolf Pfaendler, Hans-Ulrich Schmidt

**Affiliations:** 1 Department of Chemistry, Ludwig-Maximilians University Munich, 80337 Munich, Germany; 2 Department of Medical Microbiology, München Klinik gGmbH, 81925 Munich, Germany; sekretariat.ilm@muenchen-klinik.de

**Keywords:** antimicrobial resistance, carbapenemase test, AmpC beta-lactamase, fluorescent carbapenem, avibactam, relebactam, zidebactam, nacubactam, vaborbactam, infectious therapy

## Abstract

**Background/Objectives:** In this proof-of-concept study, the objective was to evaluate the phenotypic CarbaLux combination rapid test in terms of guiding the therapy of infections caused by multidrug-resistant Gram-negative bacteria with carbapenemase inhibitors and carbapenems, and to compare its results and practicability with standard diagnostic methods. **Methods:** In the classical CarbaLux test, a fluorescent carbapenem serves as a UV–visible diagnostic surrogate for clinically used carbapenem antibiotics. When exposed to extracted carbapenemases from bacterial colony growth on agar plates, fluorescence rapidly disappears, showing whether monotherapy with carbapenems is possible or must be rejected. It was expected that a specific inhibitor that protects imipenem or meropenem from enzymatic deactivation during antibacterial therapy would perform the same in vitro with fluorescent carbapenem and preserve its fluorescence. The new additional CarbaLux combination test is used if the classic test is positive for carbapenemases: a classic test tube pre-dosed with fluorescent carbapenem is spiked with cloxacillin; with recently launched carbapenemase inhibitors, e.g., avibactam, relebactam, zidebactam, nacubactam, or vaborbactam; or with picolinic acid. Fourteen Enterobacterales and six *Acinetobacter baumannii* isolates were analyzed. **Results:** At fixed concentrations, the new inhibitors protected fluorescent carbapenem from bacterial KPC-mediated inactivation and partially from AmpC beta-lactamase-mediated inactivation. In addition, avibactam also effectively inhibited OXA-48-like enzymes. Cloxacillin selectively inhibited AmpC beta-lactamases extracted from *Enterobacter* complex species. Non-therapeutic picolinic acid was specific for metallo-beta-lactamases and thus identified infections by pathogens that cannot be treated with carbapenems alone or in combination. **Conclusions:** Inhibitor/fluorescent carbapenem mixtures corresponding to therapeutic inhibitor/carbapenem combinations allow us to visualize the efficacy of carbapenemase inhibitors. The in vitro results are consistent with clinical experience regarding combination therapy. Enzymatic assays provide a rapid yes/no answer for carbapenem mono- or combination therapy and offer several advantages over current carbapenemase testing methods. In contrast to PCR and lateral flow tests, which only target a selection of carbapenemases, enzymatic assays work by employing a reproducible phenotypic mechanism. They are simpler, broader in scope, and more cost-effective; they can also detect antimicrobial heteroresistance or AmpC beta-lactamase hyperproduction, which is normally undetected when performing automated antibiotic susceptibility testing. The new tests are suitable for clinical diagnosis, public health purposes, and infection control.

## 1. Introduction

The world of bacteria continues to change. Their resistance to antibiotics is increasing worldwide, posing a threat to human health and modern medicine [1]. Almost 70% of this burden is represented by multiresistant Gram-negative pathogens. Recently, the wave has also hit the most vulnerable group: in India, previous survey data [2] estimated that there were 2.35 million deaths per year for children aged 0–4 years, and neonatal sepsis—which was prevalent in the 18th century—is now common again. This has led to a high mortality rate among newborns [3,4]. Although they used to be rare in Northern Europe, multiresistant bacteria have been brought in from wars or other risk zones, and variants of *Klebsiella pneumoniae* and *Acinetobacter baumannii* spp., formerly known as “Iraquibacter,” have also found their way into German hospitals [5,6].

Based on our observations in Germany, the numerous travelers returning home from vacation are significant importers of multidrug-resistant bacteria from risk areas [7]. They are usually colonized, not infected, and symptom-free. In this regard, a multivariate clinical study conducted in the US conclusively found that critically ill patients in medical and surgical intensive care units who were colonized with carbapenem-resistant bacteria, particularly *K. pneumoniae* spp., had an approximately 11-fold higher risk of becoming infected during treatment. The three-month survival rate was 44% compared to 95% for their non-colonized counterparts. Colonization was largely associated with previous antibiotic exposure [8]. This means that infections regularly arise from the patient’s own microbial flora in the intestines. Apart from hygienic reasons, screening for multidrug-resistant bacteria before or upon admission to a hospital can identify patients with a high risk of infection and lead to the early optimization of antibiotic treatment.

High resistance is mainly due to the production of bacterial enzymes, i.e., carbapenemases, which rapidly inactivate all beta-lactam antibiotics [9]. The detection of resistance enzymes is important for selecting an appropriate infectious therapy. This can be challenging, especially when heteroresistant and AmpC-overproducing bacteria are involved.

A major drawback in the treatment of difficult-to-treat infections arises from heteroresistant bacteria, which are often classified as sensitive in routine antibiograms but still produce carbapenemases [10]. Such strains regularly contain very few resistant mutants hidden in a large and apparently inconspicuous bacterial population. They are selected by growth in antibiotic media in vitro [11] and during antibiotic therapy in vivo [12]. In a study of 211 patients, the incidence during antibiotic therapy and the risk factors associated with colonization/infection by carbapenem-heteroresistant *A. baumannii* were similar to those described for isolates with complete carbapenem resistance [13].

Although heteroresistant bacteria were described as early as 1947 [14], it is only now suspected that misleading diagnostic tests [15] have led to the selection of new resistant pathogens and, over the long term, to ineffective antibiotics and recurrent and/or untreatable infections. Interestingly, from an etiological point of view, the largely underestimated heteroresistant group has been interpreted as being an intermediate in the journey to full resistance [16]. This hypothesis was also confirmed in a case report [17].

In contrast to completely resistant strains, heteroresistant bacteria can rapidly suppress or retain resistance determinants when sub-cultured in antibiotic-free media [10,17,18]. A recent study found that, of 766 bacterial–drug combinations tested, 27.4% showed heteroresistance, mainly of the ominous unstable type [19]. Low apparent MICs, frequently in the susceptible range, make the detection of heteroresistant species very difficult [20].

Another important issue in clinical decision making arises from heteroresistant Enterobacterales, particularly *E. cloacae* and *Klebsiella aerogenes* spp., which can hyperproduce AmpC beta-lactamases that inactivate cephalosporins and carbapenems and contribute to clinical resistance. They often appear susceptible by antibiogram, but resistance can develop as early as a single day after drug initiation [21]. In a three-year cohort study of 228 cases of bacteremia, AmpC beta-lactamase-producing *Enterobacterales* were the second highest risk factor for inappropriate therapy. Patient mortality (14/59) was even higher than that for methicillin-resistant *Stapylococcus aureus* (MRSA) infection (11/40) [22]. It has been suggested that this mechanism may even create a resistance problem that is more important than acquired carbapenemases [23]. In a recent Taiwanese study, 88 imipenem (IPM)-resistant *Escherichia coli* and *K. pneumoniae* isolates had no detectable carbapenemase, but 85 produced AmpC and/or ESBL beta-lactamases [24].

### 1.1. Treatment of Infections by Combination Therapy

The growing resistance of Gram-negative bacteria has prompted researchers to seek new solutions. To combat bacterial resistance and/or heteroresistance to carbapenems, several new potent carbapenemase inhibitors have recently become available, e.g., avibactam (AVI), relebactam (RELE), zidebactam (ZIDE), nacubactam (NACU), and vaborbactam (VABOR) [25]. Their combinations with cephalosporins or carbapenems are used to treat urinary tract, respiratory tract, and blood infections.

With the exception of ZIDE, these new inhibitors have little or no antibacterial effect on their own [26]. As compared to the earlier clavulanate/amoxycillin combination, the in vitro spectrum of activity in combination with beta-lactam antibiotics is broader and also extends to KPC-producing and, in some cases (AVI/ceftazidime), OXA-48-carbapenemase-producing *K. pneumoniae* strains [27]. However, none of the new combinations were able to overcome the significant bacterial resistance imposed by metallo-beta-lactamases (VIM and NDM) or by OXA-23 carbapenemase of *A. baumannii* [28,29], although a beta-lactam enhancer role of ZIDE/cefepime was demonstrated in an *A. baumannii* OXA-23 mouse infection model [30]. A new in vitro study performed in Taiwan reported a significant increase in IPM/relebactam resistance in urinary tract infections caused by IPM non-susceptible organisms [31].

The scope of combination strategy is limited and does not extend to the plethora of multiresistant Gram-negative bacteria in general. In conclusion, the new inhibitor/carbapenem compositions are narrow-spectrum antibacterial agents. Hence, uncontrolled therapeutic use of these combinations increases the risk of clinical failure and, in the long term, the development of even more serious resistance. Whenever empiric therapy is avoidable, they should be used after isolation and careful in vitro testing of the causative pathogens.

Compared to other microscopic organisms, bacteria are complex creatures, and their resistance to antibiotics and impacts on human health alone have prompted scientists to take a closer look at the diverse mechanisms of bacterial life. In the context of therapy, it is important to know that antibiotics and beta-lactamase inhibitors have two different targets. In Gram-negative species, antibiotics target penicillin-binding proteins anchored to the peripheral bacterial membrane. On the other hand, beta-lactamase inhibitors largely affect soluble periplasmic beta-lactamases, although extracellular release can also occur [32]. Therefore, alterations of the penicillin binding proteins and the production of carbapenemases should be considered as two different features resulting in the in vitro resistance/non-susceptibility to carbapenems in discordant scope. Particular Gram-negative species can have very low MICs and produce large amounts of carbapenemases or be resistant to carbapenems without detectable production [33]. Apart from systematic shortcomings, susceptibility tests to antibiotics alone or in combination with inhibitors do not predict therapeutic success in every situation, as clinical resistance often develops during the course of antibiotic therapy [34].

### 1.2. Current Methods for Detecting Carbapenemases and/or Bacterial Resistance

#### 1.2.1. Molecular Assays

Molecular assays (PCR tests) analyze the genetic materials of particular bacteria with high specificity to addressed genes. They do not provide direct information about the effect of inhibitors but allow us to characterize the most abundant carbapenemase genotypes. However, limited by primer sequence, molecular assays can detect only relatively few types of addressed carbapenemases. Consequently, a negative PCR test result does not rule out the production of carbapenemases in general, due to the immense number of genetic variants [35]. Less abundant or yet-unknown carbapenemases might be missed [36]. Moreover, a molecular assay for *bla*AmpCs genes would not be conclusive because (with the exception of *K. pneumoniae*) it always detects the *bla*AmpCs intrinsically located in the chromosomes of most Gram-negative genera, including those of non-resistant bacteria [37]. Molecular assays are very sensitive and require ca. one to four hours for completion.

#### 1.2.2. Lateral Flow Tests (LFTs)

Immunochromatographic tests use plastic cartridges with test strips coated with up to five antibody conjugates specific for abundant beta-lactamases or carbapenemases [38]. In contrast to PCR, they target proteins. Bacteria collected from a culture plate are dispersed in an extraction buffer and aliquots are added to the cartridges. A positive reaction can be read from colored bands after ca. 15 min. Unfortunately, like PCR, LFTs cannot provide a direct preview of the reactivity of the enzymes. Again, another remaining issue is the plurality of existing beta-lactamases. The bacteria hyperproducing the numerous different AmpC beta-lactamases largely remain undetected. An evaluation of five new lateral flow immunoassays revealed that all produced more than 10% false negative results for carbapenemases when a broad selection of 152 carbapenemase-producing Enterobacterales was investigated [39].

A different LFT panel is required for OXA-23-like carbapenemases of *A. baumannii* [40]. Therefore, at least two LFT kits with two different extraction reagents are required to investigate an unknown species. On the other hand, when the species is characterized, e.g., by MALDI TOF MS (Matrix Assisted Laser Desorption Time-of-Flight Mass Spectroscopy), if available, a single LFT kit can be valuable as a rapid confirmatory tool. A positive proof is conclusive, while a negative result is less meaningful and excludes only the targeted carbapenemases, which does not in itself justify mono- or combination therapy with carbapenems. An advantage of LFTs is the simple and rapid identification of the type of carbapenemase in positive tests.

Antibiotic resistance is a multifactorial phenomenon, and genotype-to-phenotype extrapolations are not straightforward due to the variety of gene expression and polymorphism of beta-lactamases [41]. Consequently, negative results of PCR tests should at least be supplemented by functional phenotypic methods for detecting carbapenemases and susceptibility tests [42]. This also holds for negative (phenotypic) lateral flow test results, due to the unaddressed carbapenemases.

#### 1.2.3. The Carbapenemase Inactivation Method (CIM)

In the phenotypic CIM, bacteria collected from an agar plate are incubated at a high inoculum in a test tube with an added meropenem disk for two hours. The disk is then examined on a separate agar plate inoculated with the meropenem-susceptible indicator strain, *E. coli* ATCC 25922. After 18–24 h incubation, a small or absent zone of inhibition is indicative of the inactivation of meropenem (MEM) and the presence of carbapenemase [43]. Two variants, the mCIM [44] and eCIM, use broth instead of water and extend the exposure of MEM to four hours. The eCIM additionally uses EDTA (ethylenediamine-tetraacetate) at a high concentration, thus inhibiting metallo-beta-lactamases specifically [45]. Identification is possible when also considering the result without EDTA. The CIM and their variants are simple, cost effective, and reliable, and they target all carbapenemases with a sensitivity of ca. 97% [39]. Limitations are the additional overnight culture, the effort, and handling time. Moreover, a CIM for testing the new beta-lactamase inhibitor/carbapenem combinations is not available. Simultaneous exposure of multidrug-resistant bacteria and *E. coli* ATCC 25922 on the same agar plate at a high meropenem concentration could pose a potential long-term risk for the CIM through horizontal transfer of unknown resistance determinants into the indicator strain [46].

#### 1.2.4. MIC Tests in Liquid Media and the Role of MALDI TOF MS

Alternatively, susceptibility can be assessed by MIC tests on bacteria originating from a primary culture plate. In standard testing, carbapenems or inhibitor/carbapenem combinations at graded concentrations are dissolved in culture broth and spiked with a low bacterial inoculum. After 16 to 20 h of incubation, growth, i.e., turbidity, is controlled by vision, with the MIC being the lowest antibiotic concentration that prevents growth. Automated broth microdilution tests are performed in 96-well plates, pre-dosed with the appropriate amounts of antibiotics. Likewise, the automated VITEK-2 tests work with plastic cards having 64 smaller cavities, but a higher bacterial inoculum is used. Turbidity is measured in 15 min intervals, the experimental data are compared to empirical data collected from the same types of bacteria, and the final MIC is calculated using software. Apart from turbidity measurement, the VITEK method requires the identification of the investigated species, which is usually determined by MALDI TOF MS. Forty years after its introduction, when resistance was almost non-existent, automated microdilution technology still dominates the daily routine in microbiology laboratories worldwide. It is reliable for pathogens with an essentially uniform population but not for multiresistant bacteria, which are often heteroresistant and/or produce two or more carbapenemases [47]. Among individual automated test methods, large differences and substantial errors were reported for susceptibility tests for carbapenemase-producing *Klebsiella pneumoniae* isolates [48], and VITEK-2 MIC results deviated substantially from those of standard broth microdilution tests [49]. False susceptibility has also been reported from South Africa, an area with a predominance of *K. pneumoniae* OXA-48 carbapenemases. Up to 67% of such isolates were incorrectly identified as IPM- and/or MEM-susceptible via automated susceptibility testing, demonstrating that confirmed carbapenemase-producing isolates do not present as possible carriers of carbapenemases using diagnostic methods [50]. A previous study in mice infected with OXA-48 producers showed that ETP and IPM failed to cure the rodents, although the MIC values were low [51].

A major drawback of microdilution assays is that heteroresistant subpopulations form at a very low frequency of ca. 10^−5^ to 10^−7^ [20,52,53]. In these assays, typically only ca. 10^4^ to 10^5^ cells are analyzed in each well. Therefore, the critical resistant mutants are practically absent in the samples. Logically, in the absence of other cell wall-dependent resistance factors, the dominant susceptible population cannot grow to turbidity in the presence of effective concentrations of antibiotics during overnight incubation, simulating false-positive susceptibility for the entire cell population, which can lead the physician to unknowingly prescribe the wrong antibiotic. In Spain and Greece—i.e., countries with high antimicrobial resistance—most *A. baumannii* isolates, which are heteroresistant to MEM, were determined as MEM-susceptible by automated microdilution tests, which led to fatal treatment failures [13,54].

In general, the condition in broth microdilution does not mirror the situation in the human body during long-term therapy. In geographical areas where microbes still have few determinants of resistance, the systematic shortcomings may have been less obvious and were easily overlooked. Nevertheless, the recent knowledge and high abundance of heteroresistant bacteria have opened a new view. It limits the results of the MIC test as the sole criterion for correct antibiotic treatment [19]. Many physicians are reluctant to make important decisions based solely on automated broth dilution MIC tests [55], and some laboratories still use more proven agar plate methods and hand readings [48,56].

#### 1.2.5. Agar Diffusion Susceptibility Tests

The effective inoculum level on an agar plate (evaluable fraction in a 20 mm zone) is about one hundred times higher than that in a 100 µL well, potentially improving the sensitivity for detecting resistance patterns on agar media compared to liquid tests. Bacteria grown on agar are visible to the eye and provide a simple, robust, and meaningful indicator. The plate cultures can be stored at 5 °C for several days and re-analyzed.

**Figure 1:**
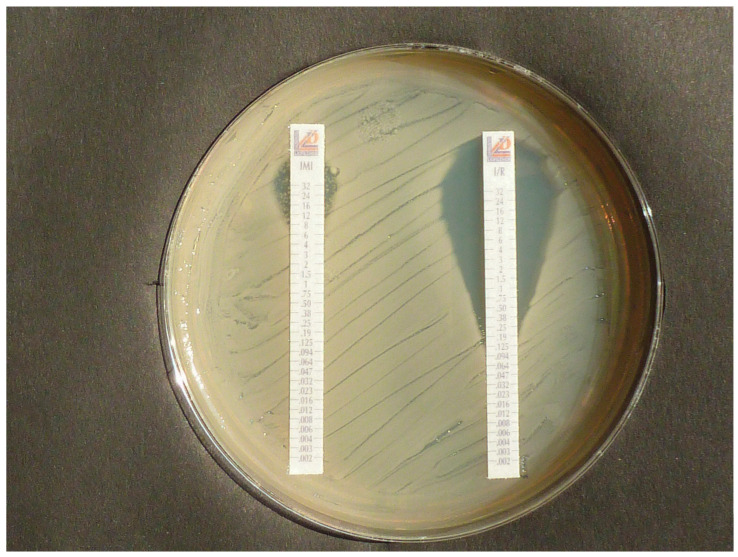
Example of MIC test strips on Mueller–Hinton agar plate showing small inhibition zone, i.e., full resistance of KPC-producing *K. pneumoniae* to IPM (**left**), and large zone, i.e., susceptibility to IPM/RELE (**right**).

The agar-based methodology may also be useful for testing new inhibitor/carbapenem or inhibitor/cephalosporin combinations. After cultivation, positive inhibition is shown by a significant increase in the inhibition zone of the combination compared to that of the antibiotic alone (Figure 1).

**Figure 2:**
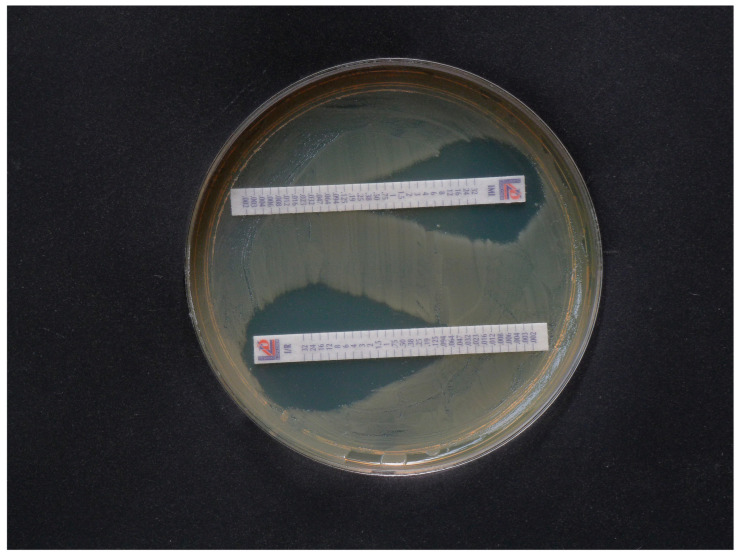
Example with MIC test strips indicating apparent susceptibility to IPM (**top**) and true susceptibility to IPM/RELE (**bottom**) of heteroresistant *E. kobei*, hyperproducing AmpC-beta-lactamase.

However, the antibiogram of the composition is often similar to that of carbapenem alone (Figure 2). In a recent in vitro study with clinical isolates of *E. coli* and *S. enterica*, heteroresistance selected by the antibiotics was not detected through the disk diffusion susceptibility test [57]. This suggests that large and distinct zones may be misleading as the sole criterion.

A more reliable assessment of heteroresistance is based on the appearance of separate colonies in the inhibition zone [11], provided that the relative rate of the resistant subpopulation is large enough (ca. > 10^−5^) to allow a few resistant mutants to grow into visible colonies in the inhibition zone of disk diffusion or gradient tests.

A multivariate analysis study conducted with 211 patients in Spain, where 21–24% of *A. baumannii* spp. showed heteroresistance inducible by carbapenems, found that isolated *A. baumannii* variants that produced single colonies in the inhibition zone of IPM or MEM were slightly more infectious than those without zones. Patient mortality was equal among in vitro fully resistant and apparently susceptible (heteroresistant) strains, but did not depend on colony growth in the inhibition zones of agar plates [13]. This indicates that differentiating heteroresistant and resistant strains might not be conclusive to guide antibacterial therapy with carbapenems.

MIC test strips allow for convenient reading of two MICs per plate, whereas the disk method can test up to six substances. Susceptibility to individual antibiotics is determined from the zone diameter, and clinical cutoff values are also available in tables (EUCAST. Clinical breakpoint table 2025) [58]. Both test procedures need additional overnight culture. An advantage of the strips is the direct reading of MICs, while a limitation is the higher costs compared to that of the disks.

### 1.3. The Two CarbaLux Tests for Detecting Carbapenemases

The operating principle and speed of the CarbaLux tests were illustrated above by real-time video. Previously, the same *E. coli* OXA-48 isolate characterized by PCR inactivated MEM appropriately, but IPM was inactivated more than ten times faster than CF. Remarkably, despite the very high carbapenemase activity, the heteroresistant isolate appeared susceptible to IPM, MEM, and ertapenem (ETP) with MICs of 2, ≤0.25, and ≤0.5 µg/mL by antibiogram.

#### 1.3.1. The Classical CarbaLux Test for Carbapenemases

Like the carbapenem inactivation method (CIM), the CarbaLux test works by enzymatic inactivation of a carbapenem. Instead of MEM or IPM, a fluorescent carbapenem—namely, carbapenem F (CF)—is used, which makes it possible to monitor the inactivation process directly by vision, according to the decay of fluorescence. The test specifically detects all extracted carbapenemases and hyperproduced AmpC beta-lactamases. In contrast to the CIM, the CarbaLux test does not require an additional overnight culture of an indicator strain and usually provides a result within a few minutes.

#### 1.3.2. The New CarbaLux Combination Assay

It makes sense to carry out the combination assay when positive carbapenemase activity was already detected in the classic screening test with the result of a dark (non-fluorescent) tube, caused by an extracted carbapenemase or a hyperproduced AmpC beta-lactamase. Alternatively, both tests can be run in parallel. The combination assay differs from the classical CarbaLux test for carbapenemases in that the extract of a bacterial isolate is exposed to a composition of CF and specific carbapenemase inhibitors. It allows us to investigate the synergy of the two components. The activity of the inhibitor is evaluated by comparing the decay or the resulting fluorescence end value with that of the uninhibited process, i.e., the result of the classic CarbaLux test. In the combination test, a fluorescent tube after 30 min or 1 h or substantially retarded decay of fluorescence indicates the inhibition of carbapenemases and/or hyperproduced AmpC beta-lactamases, while a dark tube indicates an ineffective inhibitor.

Originally, the inhibitor used was cloxacillin (CLX), which is specific for hyperproduced AmpC beta-lactamases that also deactivate carbapenems [59]. When testing hyperproducing strains, in the absence of carbapenemases, the fluorescence of CLX/CF was preserved. On the other hand, a dark tube indicated that the investigated bacteria produced a carbapenemase not inhibited by CLX. Thus—and in contrast to genotypic assays or lateral flow tests—hyperproduced AmpC beta-lactamases could also be specifically identified. Based on our experience with the cloxacillin/CF combination test, the same principle was applied with five new and two older inhibitor/CF compositions, thus expanding the scope of the test. Twenty representative hospital strains were investigated.

When developing the new CarbaLux combination test, the problem was to find a ratio of inhibitor to CF that could be applied to all bacteria, since even within a given class of carbapenemases, enzyme reactivity and gene expression vary greatly among individual species. Therefore, the strain with the highest carbapenemase activity, KPC-producing *K. pneumoniae*, was selected for the experimental setup in the expectation that less reactive variants would also be inhibited and pass the combination test. Of course, the amount of inhibitor also affects the test result in that a higher inhibitor concentration leads to higher inhibitory activity. We aimed for complete in vitro inhibition, i.e., full preservation of fluorescence checked after one hour at 36 °C. First, avibactam/CF was investigated. For diagnostic purposes, a ratio of avibactam/CF of 3:1 was found to be suitable to provide full inhibition of KPC in *K. pneumoniae*. For the four other new corresponding compositions, we chose the same 3:1 inhibitor/CF ratio. This enables a meaningful comparison of the inhibition activities. On the other hand, picolinic acid (PIC) was far less effective and required a 150:1 ratio, which limits its use to diagnostic purposes.

## 2. Material and Methods

### 2.1. Bacterial Strains and Culturing

Bacterial strains were obtained from the collection of München Klinik GmbH, München, Germany. Carbapenemase genes were confirmed via PCR, and AmpC-hyperproducing isolates were characterized phenotypically through the CarbaLux combination test with cloxacillin. Tested strains were *K. pneumoniae* KPC (2), *K. pneumoniae* OXA-181 (1), *K. pneumoniae* OXA-48 (1), *K. pneumoniae* VIM (1), *K. pneumoniae* ESBL (1), *E. coli* OXA-48 (2), *E. coli* NDM (1), *E. coli* ESBL (1), *A. baumannii* OXA-23 (3), *A. baumannii* OXA-40 (1), *A. baumannii* OXA72 (1) *A. baumannii* OXA-23/NDM (1), *E. cloacae* hyperprod. AmpC (1), *K. aerogenes* hyperprod. AmpC (1), *E. kobei,* hyperprod. AmpC (1), and *E. ludwigii* hyperprod. AmpC (1). *E. coli* ATCC 25922 served as a carbapenemase negative control strain. For a balanced selection, the following criteria were taken into account: 1. availability, 2. diversity of species, 3. a positive PCR test for at least one carbapenemase gene, 4. a positive classical test for carbapenenemases or hyperproduced AmpC beta-lactamase, 5. relevance and frequency in nosocomial infections, 6. diversity of carbapenemases, 7. different genera, 8. more than one carbapenemase, and 9. ESBL (non-carbapenemase)-producing strains. MIC results were not taken into account for the selection. *Pseudomonas aeruginosa* spp. was not included in this study as some strains were self-fluorescent and potentially interfered with fluorescence testing. They require additional special handling, as published [59].

Cultures on Columbia agar plates with 5% sheep blood (product No. PB 5039 A from Thermo Scientific, Waltham, MA, USA) provided the best results. Inoculated plates were incubated for 15 to 18 h at 37 °C.

### 2.2. Materials and Preparation of Stock Solutions and Stability of Reagents for the CarbaLux Test

Dry dimethyl sulfoxide (DMSO) from Sigma Aldrich, product number D5879, was used to prepare the stock solutions, and 99% picolinic acid (PIC), product number 11467796, was obtained from Fisher Scientific (Hampton, NH, USA). Carbapenem F (CF), the original extraction reagent, and the PIC extraction reagent containing picolinic acid were obtained from CarbaLux GmbH, 82544 Egling, Germany. Cloxacillin sodium, originated from MP Biomedicals^TM^, was purchased from Fisher Scientific, product No. 11472072. Avibactam sodium, relebactam, zidebactam, nacubactam, and vaborbactam were obtained in milligram quantities from MedChem Express Europe (eu.sales@medchemexpress.com). Additionally, 0.5% (weight to volume) stock solutions were prepared by dissolving 1 mg of the carbapenemase inhibitors in 200 µL of dry dimethyl sulfoxide. All stock solutions were stored at minus 20 °C. Appropriate aliquots were withdrawn by a microliter Hamilton precision syringe after the refrigerated glass containers had been warmed by hand. The stock solutions were very briefly exposed to room temperature and immediately refrozen. Their stability was checked by the CarbaLux inhibition test. A 0.2% stock solution of CF in dry DMSO was prepared accordingly. Storage at minus 20 °C for several months with repeated short thawing did not measurably reduce the activity of the stock solutions. The quality of the CF and CLX/CF tubes was assessed under a UV laboratory lamp at 310 nm. The active dry substance shows an intense blue fluorescence, turning yellow-green upon dissolution in aqueous media. The PIC buffer (100 µL) was applied in undiluted form. Like the original aqueous extraction reagent, the PIC buffer was stable for 1 year at room temperature. The shelf life of the tubes pre-dosed with CF or CLX/CF, also obtained from CarbaLux GmbH, is more than one year at room temperature when protected from light. The container should be resealed immediately after removing the required number of tubes.

### 2.3. Measurement of Decay of Fluorescence

**Figure 3:**
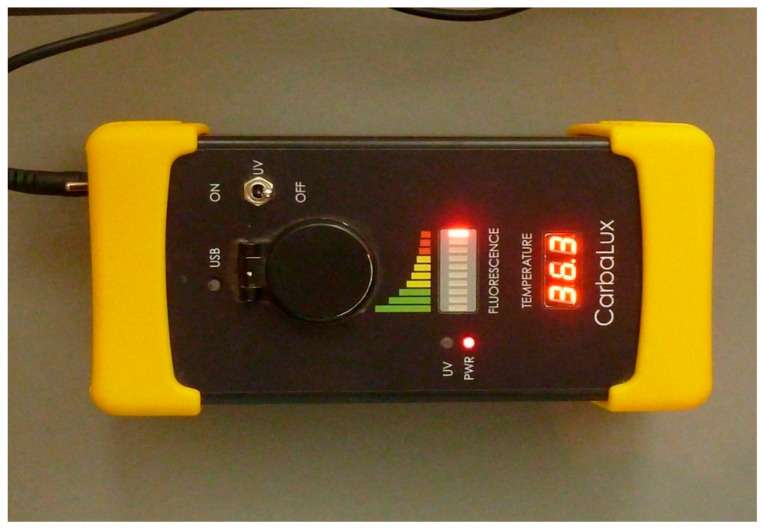
Portable table-top fluorometer allows for measurement of CF fluorescence in daylight.

Fluorescence was measured visually in the dark under a 2-tube UV hand lamp, UV-12 M (2 × 6 W), wavelength 310 nm, from Herolab GmbH, Wiesloch, Germany, Wiesloch, Germany. Alternatively, a simple table-top fluorometer (Figure 3) can also measure CF fluorescence in daylight. It can be read from an LED array, which is divided into green/yellow/red and indicates the strength of the emitted fluorescent light. The gross signal levels typical of all tests can be recorded manually or when connected to a computer. Cutoff values are not required, as the emitted CF fluorescence for all carbapenemase-positive isolates disappears completely within minutes (red LED area) and remains stable for hours for carbapenemase-negative isolates or when carbapenemases are inhibited (green LED area).

### 2.4. Protocols of the CarbaLux Combination Test

#### 2.4.1. Test Without Pre-Exposure to Inhibitor

A plastic tube pre-dosed with CF was labeled with a permanent marker and CarbaLux extraction buffer (100 µL) was added, followed by 0.5% inhibitor stock solution (2.4 µL) in DMSO by means of a Hamilton precision syringe. Subsequently, a full 3 mm plastic loop, with approximately 5 to 7 mg of wet bacteria, was collected from the agar surface and manually dispersed in the obtained liquid. The tubes were closed and the mixture was further processed at 1250 rpm to a homogeneous suspension by a vortexer. A preferred and simpler alternative was to disperse the pellet in extraction buffer by subjecting the tube and the loop to an ultrasonic water bath at room temperature for 1 min, followed by washing down residual bacteria from the tube wall with the loop. The closed tubes were left at room temperature for 15 min to allow complete extraction of the crude bacterial proteins, including the carbapenemases. The tubes were then kept in a heating block of 36 °C. The residual amount of CF was checked after 3 min, 30 min, 1 h, and optionally 2 h by vision under a UV laboratory lamp at 310 nm. The tubes should remain under the lamp only for the short periods of measurement. The level of yellow-green fluorescence was recorded in comparison to that of a negative control, i.e., a tube without bacteria or with *E. coli* ATCC 25922. A more reliable result can be read when two tests are run in parallel on a particular test strain with and without added inhibitor (see real-time video), allowing us to estimate the activity of the enzymes and of the inhibitor simultaneously. Retained fluorescence indicated an effective inhibitor, and a dark tube was consistent with an inactive inhibitor. For the assessment of metallo-beta-lactamases, the prefabricated PIC extraction medium (100 µL) was used instead of the classical CarbaLux extraction buffer. PIC can only serve as a diagnostic, not for therapy.

#### 2.4.2. Assay with Pre-Exposure to the Inhibitor

Full inhibition of all serine-type carbapenemases including AmpC requires the following procedure: The extraction medium (100 µL) was added to an empty tube (devoid of CF), followed by the 0.5% stock solution of AVI in DMSO (2.4 µL). A spherical pellet of bacteria of ca. 3 mm in diameter was then dispersed in the tube by hand or sonication as mentioned above. It was exposed at room temperature for 15 min to allow extraction and simultaneous inhibition of the enzymes. After that, a 0.2% stock solution (2 µL) of CF in DMSO was added. The suspension was vortexed, and the tube was kept in a heating block at 36 °C. Readings were made as described above.

### 2.5. Identification of Heteroresistant Isolates by Agar Diffusion Test

Heteroresistance was determined by the disk diffusion susceptibility test on Mueller–Hinton agar plates (Oxoid, Basingstoke, UK, Prod. No. PO5007A) in terms of the occurrence of isolated colonies in the inhibition zones of MEM (10 µg) and ETP (10 µg) disks and of the AVI/MEM (5 + 10 µg) combination after 20 h and 40 h incubation at 37 °C. Tests were performed following the standard method according to the protocol of American Society for Microbiology [60]. Disks were from OXOID. MIC strips were from Liofilchem/Bestbion dx GmbH, Köln, Germany.

### 2.6. Identification of the Isolates by PCR and Lateral Flow Tests

All carbapenem non-susceptible strains, selected for the CarbaLux test, were investigated for carbapenemase genes using at least one of the following molecular assays: eazyplex^®^ SuperBug CRE (Amplex Biosystems GmbH, Giessen, Germany) or Xpert^®^-Carba-R (Cepheid GmbH, Frankfurt, Germany). Addressed carbapenemase genes were *bla*_KPC_, *bla*_NDM_, *bla*_VIM_, *bla*_IMP-1_, *bla*_OXA-48_, *bla*_OXA_23, *bla*OXA-24/40, *bla*OXA-72, and in one instance *bla*OXA-181.

Lateral flow test kits, O.K.N.V.I. RESIST-5 and RESIST ACINETO, were from Coris BioConcept, 5032 Gembloux, Belgium. Tests were performed according to the procedure of the provider. The LFT results were not checked by repetition. Agar diffusion results were confirmed by a single repetition.

### 2.7. Comparative Investigations by Lateral Flow Test and Agar Diffusion Test

In addition to the CarbaLux tests, PCR, lateral flow tests (LFTs), and agar diffusion tests were performed. Bacteria were from the same overnight cultures on Difco Columbia agar with sheep blood.

## 3. Results

### 3.1. CarbaLux Test Results

The results from twenty clinical isolates and a negative reference strain with seven carbapenemase inhibitor/carbapenem F combinations are summarized in Table 1. The experiments were carried out without pre-exposure of the extracts to the inhibitors, i.e., the collected bacterial pellet was added to the inhibitor/CF composition last. The inhibitory activity was as expected for the different carbapenemase types, previously identified by PCR assays. All data were confirmed through at least two repeated experiments.

The values in Table 1 give a rough overview of the in vitro activities of the inhibitors. In agreement with previous results with avibactam and relebactam (MK-7655) [61], the five new carbapenemase inhibitors tested only worked with certain extracts with serine-type enzymes, e.g., KPC carbapenemases or partially with hyperproduced AmpC beta-lactamases, while picolinic acid alone was a specific inhibitor for metallo-beta-lactamases. Avibactam had the broadest spectrum and also inhibited extracted OXA-48 and OXA-181 carbapenemases in vitro. As previously stated, vaborbactam does not provide additional protection of carbapenems from class C/AmpC beta-lactamase [62]. Cloxacillin specifically inhibited AmpC beta-lactamase.

Without an added inhibitor, the KPC-, OXA-48-like-, VIM-, and NDM-carbapenemase-producing bacteria were the most reactive with instantaneous removal of fluorescence when the bacterial pellet was mixed in. On the other hand, the *A. baumannii* spp. producing an OXA-23 carbapenemase were less reactive but still provided a positive classical CarbaLux test result for carbapenemase by complete removal of fluorescence in less than 15 min at 36 °C.

The two ESBL strains and *E. coli* ATCC 25922 were carbapenemase-negative and always provided stable fluorescence. This agrees with the general susceptibility of ESBL-producing strains to carbapenem antibiotics [63]. Expectedly, the fluorescence of CF with the extracted *E. coli* ATCC 25922 strain was also stable for >24 h at 36 °C.

Table 2 provides an overview of the influence of the CF/AVI ratio in the CarbaLux inhibition test and the increased inhibition by pre-exposure to AVI.

At low concentration, AVI inhibited the extracted KPCs and three of four hyperproduced AmpC enzymes (derived from hyperproducing *E. cloacae* complex strains) but not the more reactive extracted carbapenemases OXA-48 and OXA-181. These results can be explained by the established kinetic parameters [64,65], which confirm the significantly higher apparent catalytic efficiency (*k*_cat_/*K*_m_) of KPCs in hydrolyzing IPM and the preference of avibactam to inhibit KPCs compared to OXA-48 [66].

On the other hand, at a higher concentration of AVI, the more reactive OXA-48 or OXA-181 enzymes were also inhibited, but not (OXA-23, OXA-40, or OXA-72, which are exclusively found in *A. baumannii*).

A modification of the protocol allowed more efficient inhibition of all serine-type beta-lactamases, including the OXA-23, OXA-40, and OXA-72 variants, by pre-exposing the collected bacteria in the extraction buffer to AVI alone for fifteen minutes at room temperature and then adding the appropriate amount of CF in DMSO solution. During the pre-exposure period to AVI, the enzymes are extracted concomitantly. Alternatively, the pre-exposed bacterial suspension can be transferred into a CF tube. An isolate with OXA-23 and NDM carbapenemases provided reduced fluorescence or a dark tube within 15 min at 36 °C when pre-exposed to AVI or PIC alone, respectively (Table 2).

The modified protocol was also applied to the *A. baumanni* OXA-23/NDM isolate. Double inhibition provided a fluorescent tube when the bacteria were pre-exposed to AVI at room temperature in the PIC buffer and CF was added after 15 min. Fluorescence then remained stable for more than 6 h at 36 °C.

### 3.2. Results from PCR and Lateral Flow Tests

The LFT detected all carbapenemases in fourteen isolates, gave a correct negative result for two ESBL strains, and fulfilled all expectations based on PCR results for the corresponding carbapenemase genes. *A. baumannii* OXA-72 carbapenemase was recognized as a faint band on the Carba Acineto chromatography strip, reporting as OXA-58 carbapenemase, which was considered positive according to the provider’s recommendations. Hyperproduced AmpC beta-lactamases and ESBL beta-lactamases were not addressable via the LFT.

### 3.3. Summarized Data from Standard Methods and the CarbaLux Tests

Table 3 shows the results from the twenty clinical isolates and from the beta-lactamase-negative reference strain *E. coli* ATCC 25922.

### 3.4. Features and Performance of Three Standard Methods and the CarbaLux Test

In Table 4, the current diagnostics methods are compared with the CarbaLux tests. The data provide information about practicability and costs. The latter are estimates from Germany. They might be different in other countries.

### 3.5. Proof of Heteroresistance and Results of Agar Diffusion Tests

**Figure 4:**
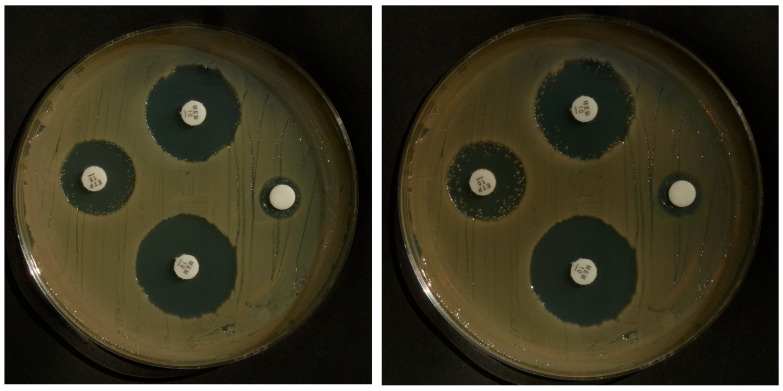
Effect of incubation time to detect heteroresistance by occurrence of isolated bacterial colonies in inhibition zones. Example with No. 3 strain OXA-48-producing *K. pneumoniae:* MEM 10 µg (top), (clockwise) AVI 5 µg AVI/MEM 5 + 10 µg, and ETP 10 µg. Visible colonies in MEM zone are not detectable on agar plate after 20 h incubation (**left side**) but are after 40 h of incubation (**right side**). AVI/MEM zones (bottom) remained clear over 40 h. Both photographs were taken from same agar plate.

Heteroresistance was assessed by the occurrence of single colonies in the inhibition zone of meropenem and confirmed with isolates hyperproducing AmpC beta-lactamase (3), OXA-48 (3), and OXA 181 (1), as well as VIM (1), NDM (1) and KPC (1) carbapenemases. Isolate No. 6, ESBL-producing *K. pneumoniae*, had reduced susceptibility to MEM with the formation of single colonies, which may be due to reduced cell wall permeability. Notably, the formation of single colonies could be substantially slow, and isolates No. 3 and No. 4 took 40 h of incubation at 37 °C to provide a clear positive result for heteroresistance to MEM. The example with isolate No. 3 is shown in Figure 4. 

Heteroresistant *E. coli* isolate No. 7, producing OXA-48 carbapenemase, did not provide visible colonies in this test, even when the plate was exposed to 37 °C for forty hours. This result is consistent with previous reports of heteroresistant *A. baumannii* spp., which also did not form single bacterial colonies [18]. The carbapenemase-producing *A. baumannii* isolates (6) and *E. ludwigii* strain No. 13 were fully resistant without significant inhibition zones.

## 4. Discussion

**Scheme 1:**
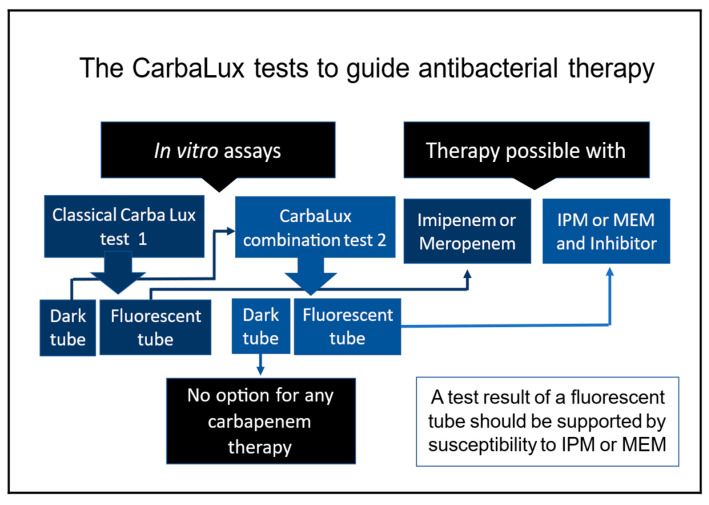
A diagnostic flowchart for converting the results of the classic CarbaLux test (1, dark blue) and the combination test (2, blue) for the appropriate clinical treatment (right) of infections by carbapenems. Since the fluorescent substrate is a carbapenem, the loss of fluorescence is due to the presence of a bacterial carbapenemase which is incompatible with monotherapy with IMP or MEM. There is still an option for combination therapy, which can be investigated by the combination test. If a carbapenemase inhibitor protects the fluorescent carbapenem in in vitro combination test 2, it can be expected that this will be the case in combination therapy with IPM or MEM. For a rapid result, both tests 1 and 2 (without and with carbapenemase inhibitor) should be performed simultaneously. A positive decision in favor of carbapenem therapy should be supported by demonstrating the susceptibility of the isolate to carbapenems to rule out the possibility of non-carbapenemase resistance.

Scheme 1 presents a flowchart for implementing the results of in vitro CarbaLux tests for clinical decision making.

### 4.1. The Effect of Pre-Exposure to Avibactam

We explain the effect of pre-exposure, according to Table 2, by the higher affinity and reactivity of CF to the serine-type carbapenemases as compared to AVI. When applied simultaneously, the two components CF and AVI compete for binding to the enzyme, with the more reactive CF being rapidly deactivated. In contrast, during preincubation, the less reactive AVI has more time for complete enzyme inhibition. As a result, the synergistic effect is significantly better than in the non-incubated process. This hypothesis is supported by the broad and almost irreversible inhibition of serine-type beta-lactamases by AVI [66]. The method could also be useful for the evaluation of new carbapenemase inhibitors with a broader spectrum of activity and/or the investigation of resistance mechanisms.

### 4.2. Comparison with Standard Test Methods

Our results (Table 3) from the lateral flow tests (LFTs) and PCR tests for the identification of carbapenemases showed complete agreement with the classical CarbaLux test, which detected carbapenemase in the same fourteen isolates and was negative for the two ESBL producers. On the other hand, this study shows the shortcomings of PCR and LFT, i.e., the false negative results when it comes to detecting carbapenem resistance, which is mediated by the overproduction of AmpC beta-lactamase and is widespread in *Enterobacter* complex species.

Predictably, such a concordance could not be experimentally determined between the growth-based agar diffusion test for meropenem and the classical CarbaLux test for carbapenemases, which aim at different targets. Neither a low susceptibility to MEM (i.e., an inhibition zone diameter below the cutoff value of 16 mm) nor individual colonies in the inhibition zones correlated significantly with carbapenemase production, and ESBL production did not significantly correlate with the cutoff value ≥ 22 mm. In particular, most *K. pneumoniae* and *E. coli* isolates producing an OXA-48-like carbapenemase and AmpC-producing Enterobacter complex species exhibited large zones of inhibition with MEM alone. On the other hand, as suggested by EUCAST [58], small diameters (indicating complete resistance) or visible colonies in the inhibition zones (indicating heteroresistance) collectively agreed better (90%) with carbapenemase production. The observed increase in inhibition zone diameter was often marginal, only a few mm, which is hardly conclusive and reproducible.

In practice, however, the number of visible heteroresistant colonies can be scarce and easily overlooked. Prolonged incubation to 40 h was required to detect few mutant colonies in the meropenem and ertapenem inhibition zones of, e.g., isolate No. 3 (Figure 4) and isolate No. 8, which increases the time to result substantially, making this method for detecting heteroresistance time-consuming and difficult to interpret. With isolate No. 7, visible colonies could not be detected in the MEM zone even after 40 h of culturing. This result is consistent with previous reports of heteroresistant *A. baumannii* spp., which did not always form single bacterial colonies [13].

**Figure 5:**
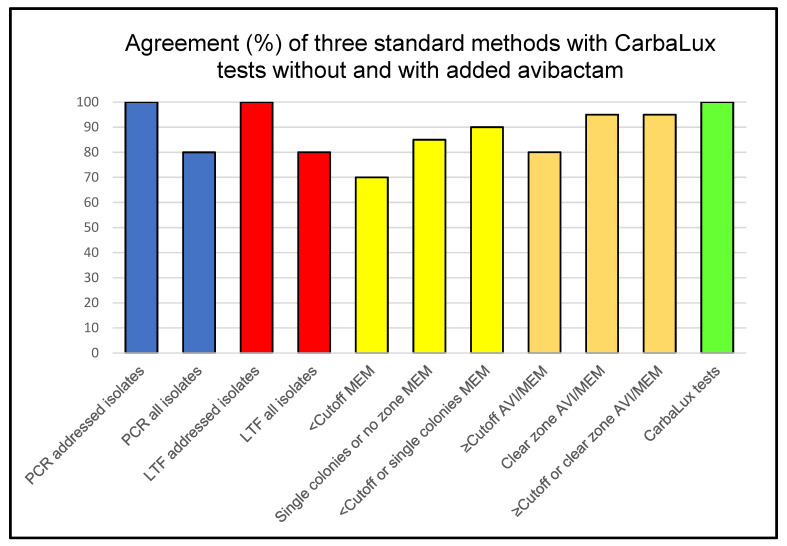
Potential agreement of twenty (fourteen addressed by PCR and LFT) results according to Table 3 from polymerase chain reaction (PCR, blue columns), lateral flow test (LFT, red columns), and agar susceptibility test (yellow columns) to meropenem with the results of the classical CarbaLux test (green column) for detection of carbapenemases and hyperproduced AmpC beta-lactamases in Gram-negative bacteria. The orange columns refer to agar susceptibility tests of avibactam/meropenem combination, in comparison with twenty results of the CarbaLux inhibition assay with avibactam/CF (green column). The cutoff of the AVI/MEM combination was arbitrarily set to S ≥ 22 and R < 22. The plates were read after 40 h of incubation at 37 °C. The middle yellow row also includes test results without inhibition zones.

Figure 5 shows the calculated agreement of three standard methods (PCR, LFT, and agar diffusion) with the CarbaLux tests according to Table 3. 

Regarding combination testing of AVI and MEM, the best agreement (95%) with the results of the CarbaLux combination assay was found when considering clear inhibition zones of AVI/CF with or without taking increased diameters into account, demonstrating the importance of individual colonies in the evaluation of carbapenem resistance and heteroresistance by agar plate antibiograms. In conclusion, special attention should be paid to the analysis of inhibition zones to assess the clinical utility of carbapenemase inhibitor/carbapenem compositions.

**Figure 6:**
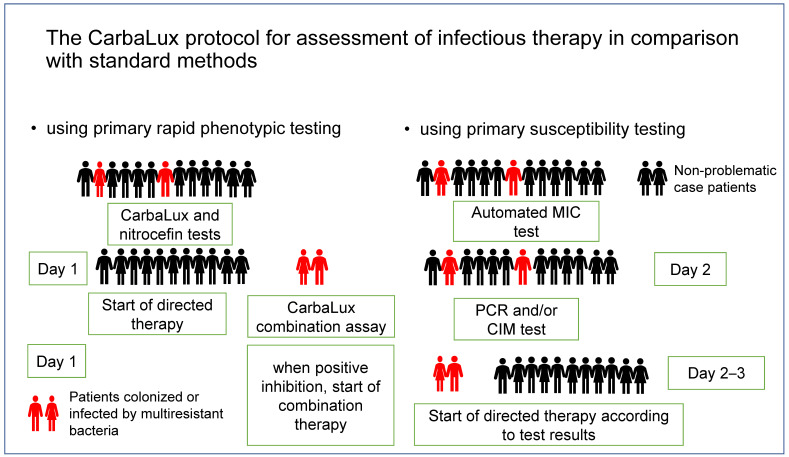
Protocol for the diagnostic identification of multiresistant bacteria and clinical therapy with beta-lactam antibiotics. The time scale is based on already completed primary agar cultures. In areas with low abundance of resistance, the majority of patients, indicated in black, are colonized or infected by non-resistant or low-resistant pathogens. The chart on the left side explains the early selection of the problematic case patients, indicated in red, and the immediate start of adequate therapy delayed by at least one day when primarily guided by MIC tests (right-side chart).

Time is an essential factor in therapeutic interventions. Logically, it makes sense to use rapid and informative methods first. In particular, the nitrocefin test [67] is a proven tool, as it detects entire beta-lactamase production and also characterizes inconspicuous strains with a negative result for beta-lactamases. On the other hand, it cannot distinguish between carbapenemases and other beta-lactamases on its own. However, in conjunction with the CarbaLux tests, it allows microbiologists and physicians to start appropriate therapy earlier, by screening out and focusing on problematic patients, i.e., those colonized or infected by multidrug-resistant bacteria. This can significantly reduce the workload and costs in clinics in all areas with or without a high burden of bacterial resistance. A protocol for rapid diagnosis is proposed in Figure 6.

### 4.3. Advantage of the CarbaLux Tests in Clinical Decision Making

- Phenotypic tests enable the gross reactivity of bacterial enzymes to be assessed.
- They provide a clear yes/no answer regarding the presence or absence of carbapenemases and enable simple visual evaluation of the results.
- The fluorescence can also be easily read with photoelectronic devices. Therefore, the test methods could potentially be automated.
- The test results are available at least one day earlier than with growth-based assays.
- The tests can also identify suspicious heteroresistant bacteria.
- Metallo-beta-lactamases can be detected by inhibition with the PIC buffer or by exclusion of all serine beta-lactamases through pre-exposure of the bacteria to AVI.
- Bacteria from primary culture plates are examined as they are regularly used for further tests, including MIC tests.
- No expensive equipment is required. The tests work with a few micrograms of CF substrate. This makes the tests inexpensive and affordable even in low-income countries.
- Due to their high sensitivity, the tests can also identify bacteria that hyperproduce AmpC beta-lactamases, which inactivate clinical carbapenems, especially IPM.
- The classic CarbaLux test enables a decision for or against monotherapy with clinical carbapenems.
- The CarbaLux combination test enables a decision for or against combination therapy with carbapenemase inhibitors and carbapenems.

### 4.4. Sensitivity and Specificity of the Classical CarbaLux Test

Full concordance with PCR and LFT results based on 14 confirmed strains showed a sensitivity and specificity of 100%, in agreement with 39 previously tested carbapenemase-positive and 19 negative partially identical tested isolates [59]. The performance of the new CarbaLux combination test could not be calculated, as there is no approved reference method that measures the activity of carbapenemase inhibitors to restore the antibacterial activity of carbapenems.

### 4.5. Scope and Limitations

A limitation of our results with the new CarbaLux inhibition tests is the relatively small number of strains tested. Broader studies should be conducted. A further limitation regarding the interpretation of agar diffusion combination tests is the lack of published clinical cutoff values for the avibactam/meropenem combination, so the values were chosen arbitrarily.

## 5. Conclusions

As there are fewer and fewer options for treating complicated infections, rapid diagnosis is crucial to curb antibiotic resistance and minimize the consequences of inappropriate therapy for patients. The simple CarbaLux tests can improve clinical decision making in cases of infection with multidrug-resistant bacteria. Routine use of rapid tests can fill a long-standing gap and contribute to more reliable diagnosis, improved antibiotic management, and correct treatment of bacterial infections.

## Figures and Tables

**Table 1:** Performance metrics of the CarbaLux combination test of twenty isolates with seven inhibitor/fluorescent carbapenem (CF) combinations. The level of fluorescence was recorded immediately after the suspension of the bacteria, after 15 min at room temperature, and then after exposure of the tubes at 36 °C for 15, 30, and 60 min and compared with the level of the non-inhibited process in each case.

	*n*	AVI	RELE	ZIDE	NACU	VABOR	CLX	PIC	Without Inhibitor
Ratio inhibitor: CF		3	3	3	3	3	10	150	
Type									
KPC	2	(2/2)	(2/2)	(1/2)	(1/2)	(2/2)	(0/2)	(0/2)	(0/2)
OXA-48	3	(3/3)	(0/3)	(0/3)	(0/3)-	(0/3)	(0/3)	(0/3)	(0/3)
OXA-181	1	(1/1)	(0/1)	(0/1)	(0/1)-	(0/1)	(0/1)	(0/1)	(0/1)
OXA-23	3	(0/3)	(0/3)	(0/3)	(0/3)	(0/3)	(0/3)	(0/3)	(0/3)
OXA-40	1	(0/1)	(0/1)	(0/1)	(0/1)	(0/1)	(0/1)	(0/1)	(0/1)
OXA-72	1	(0/1)	(0/1)	(0/1)	(0/1)	(0/1)	(0/1)	(0/1)	(0/1)
AmpC hyperprod.	4	(4/4)	(2/4)	(3/4)	(4/4)	(0/4)	(4/4)	(0/4)	(0/4)
NDM	1	(0/1)	(0/1)	(0/1)	(0/1)	(0/1)	(0/1)	(1/1)	(0/1)
VIM	1	(0/1)	(0/1)-	(0/1)	(0/1)	(0/1)	(0/1)	(1/1)	(0/1)
OXA-23/NDM	1	(0/1)	(0/1)	(0/1)	(0/1)	(0/1)	(0/1)	(0/1)	(0/1)
ESBL	2	(2/2)	(2/2)	(2/2)	(2/2)	(2/2)	(2/2)	(2/2)	(2/2)
*E. coli* ATCC 25922	1	(1/1)	(1/1)	(1/1)	(1/1)	(1/1)	(1/1)	(1/1)	(1/1)

*n* = number of tested isolates. ( ) = proportion of isolates with gross preservation of fluorescence after 1 h at 36 °C. CF = fluorescent carbapenem, AVI = avibactam, RELE = relebactam, ZIDE = zidebactam, NACU = nacubactam, VABOR = vaborbactam, CLX = cloxacillin, and PIC = picolinic acid.

**Table 2:** Phenotypic characterization of serine-type carbapenemases with compositions of AVI/CF and effect of pre-exposure to inhibitor.

	*n*	Without Pre-Exposure to Avibactam		With Pre-Exposure to Avibactam for 15 min RT
Ratio Avibactam/CF		1	3	1
Type				
KPC	2	(2/2)	(2/2)	(2/2)
OXA-48	3	(0/3)	(3/3)	(3/3)
OXA-181	1	(0/1)	(1/1)	(1/1)
OXA-23	3	(0/3)	(0/3)	(3/3)
OXA-23/NDM	1	(0/1)	(0/1)	(0/1) ^1^
OXA-40	1	(0/1)	(0/1)	(1/1)
OXA-72	1	(0/1)	(0/1)	(1/1)
AmpC hyperprod.	4	(3/4)	(4/4)	(4/4)

*n* = number of tested isolates. ( ) = proportion of isolates with gross preservation of fluorescence after 1 h at 36 °C. CF = fluorescent carbapenem. AVI = avibactam. ^1^ Reduced fluorescence after 1 h at 36 °C.

**Table 3:** Results from investigations by three standard methods and the CarbaLux tests for detecting resistance or carbapenemases in fourteen *Enterobacterales* and six *A. baumannii* isolates. To increase test sensitivity on agar plates, single colonies (SCs) were recorded after 40 h of incubation at 37 °C.

Isolate	PCR	Lateral Flow Tests CORIS RESIST-5 RESIST ACINETO		Disk Agar Diffusion Method, Inhibition Zone Diameter (mm)			CarbaLux Test with CF Time to Positive Result ^1^	CarbaLux Inhibition Assay Time While Inhibition Remains Effective with ^2^		
				MEM * 10 µg	AVI 5 µg	MEM+AVI 10 + 5 µg		AVI/CF 3:1	CLX/CF 10:1	PIC/CF 150:1
1. *K. pneumoniae* 2/2020 KPC	KPC+	KPC+	neg.	10 SC	11 CL	19 CL	1 min RT	>1 h 36 °C IN	1 min RT NO	1 min RT NO
2. *K. pneumoniae* 6/2024 KPC	KPC+	KPC+	neg.	≤6	≤6	16 SC	1 min RT	>1 h 36 °C IN	1 min RT NO	1 min RT NO
3. *K. pneumoniae* OXA-48	OXA-48+	OXA-48+	neg.	23 SC	8	25 CL	1 min RT	>1 h 36 °C IN	1 min RT NO	5 min RT NO
*4. K. pneumoniae* OXA-181	OXA-181+	OXA-48+	neg.	23 SC	≤6	24 CL	3 min RT	>6 h 36 °C IN	1 min RT NO	1 min RT NO
5. *K. pneumoniae* VIM	VIM+	VIM+	neg.	15 SC	≤6	15 SC	1 min RT	1 min RT NO	1 min RT NO	>12 h 36 °C IN
6. *K. pneumoniae* ESBL 2025	neg.	neg.	neg.	16 SC	≤6	22 CL	>12 h 36 °C neg. result	>12 h 36 °C NO	>12 h 36 °C NO	>12 h 36 °C NO
7. *E. coli* 573 OXA-48	OXA-48+	OXA-48+	neg.	22 CL	10	24 CL	1 min RT	>1 h 36 °C IN	1 min RT NO	1 min RT NO
8. *E. coli* 3/2017 OXA-48	OXA-48+	OXA-48+	neg.	22 SC	9	24 CL	1 min RT	>1 h 36 °C IN	1 min RT NO	1 min RT NO
9. *E. coli* NDM	NDM+	NDM+	NDM+	16 SC	12 CL	18 SC	1 min RT	1 min RT NO	1 min RT NO	>12 h 36 °C IN
10. *E. coli* ESBL 1	neg.	neg.	neg.	30 CL	10	30 CL	>12 h 36 °C neg. result	>12 h 36 °C NO	>12 h 36 °C NO	>12 h 36 °C NO
11. *E. cloacae* hyperprod. AmpC	neg.	neg.	neg.	24 SC	≤6	28 CL	5 min RT	>2 h 36 °C IN	>6 h 36 °C IN	15 min RT NO
12. *E. kobei* hyperprod. AmpC	neg.	neg.	neg.	24 SC	10	28 CL	1 min RT	>6 h 36 °C IN	>6 h 36 °C IN	1 min RT NO
13. *E. ludwigii* hyperprod. AmpC	neg.	neg.	neg.	≤6	10	17 CL	5 min 36 °C	>6 h 36 °C IN	>6 h 36 °C IN	5 min 36 °C NO
14. *K. aerogenes* 93 hyperprod. AmpC	neg.	neg.	neg.	14 SC	≤6	21 CL	1 min RT	>6 h 36 °C IN	>6 h 36 °C IN	1 min RT NO
15. *A. baumannii* 42 OXA-23	OXA-23+	neg.	OXA-23+	≤6	≤6	≤6	15 min 36 °C	15 min 36 °C NO	5 min 36 °C NO	15 min 36 °C NO
16. *A. baumannii* 00 OXA-23	OXA-23+	neg.	OXA-23+	8	10	12 SC	15 min RT	15 min RT NO	15 min RT NO	15 min RT NO
17. *A. baumannii* 90 OXA-23	OXA-23+	neg.	OXA-23+	≤6	≤6	≤6	15 min RT	15 min RT NO	15 min RT NO	15 min RT NO
18. *A. baumannii* OXA-40	OXA-40+	neg.	OXA-40/58+	≤6	≤6	≤6	15 min RT	15 min RT NO	5 min RT NO	5 min RT NO
19. *A. baumannii* OXA-72	OXA-72+	neg.	OXA-40/58+ weak signal	≤6	≤6	≤6	5 min 36 °C	5 min 36 °C NO	5 min 36 °C NO	5 min 36 °C NO
20. *A. baumannii* OXA-23/NDM	OXA- 23+NDM+	NDM+	OXA 23+ NDM+	≤6	≤6	≤6	15 min RT	15 min 36 °C NO	15 min RT NO	15 min RT NO
*E. coli* ATCC 25922 (reference strain.)	n.d.	n.d.	n.d.	29 CL	9	29 CL	>24 h 36 °C neg. result	>24 h 36 °C NO	>24 h 36 °C NO	>24 h 36 °C NO

^1^ Time to dark tube (positive test for carbapenemase/hyperproduced AmpC beta-lactamase in the classical CarbaLux test). ^2^ Time period to dark tube in presence of avibactam (AVI) or picolinic acid (PIC) in the CarbaLux inhibition assay without pre-exposure of the inhibitor. SC: few or several single colonies in the inhibition zone (heteroresistance) after 40 h of incubation. CL: clear inhibition zone (no heteroresistance) after 40 h of incubation. CF: fluorescent carbapenem; MEM: meropenem; AVI: avibactam; CXL: cloxacillin; IN: positive inhibition; NO: no inhibition. * Clinical breakpoints for meropenem for indications other than meningitis according to EUCAST 2025 are S ≥ 22 mm and R < 16.

**Table 4:** Performance, time expenditure, and estimated costs of selected diagnostic methods for detection of multiresistant bacteria or for carbapenemases to guide antibacterial mono- or combination therapy.

Features	Methods						
	PCR	Automated Broth Dilution	Lateral Flow Test	Disk Agar Diffusion	Carbapenem Inactivation Method CIM	CarbaLux Test	CarbaLux Test with Inhibitor
Identification of species required for result	no	yes	no/yes ^1^	yes *	no	no	no
Detection of heteroresistance	yes	no	yes	yes ^2^	yes	yes	yes
Detection of hyperproduced AmpC beta-lactamases	no	no	no	yes ^2^	yes	yes	yes
Detection of all carbapenemases	no	no **	no	no **	yes	yes	no
Identification of carbapenemase-type	yes, when addressed	no	yes, when addressed	no	no	no	yes
Semiquantitative assessment of carbapenemase activity	no	no	no	no	yes	yes	yes
Allows direct isolate-specific conclusion for mono therapy	no	only with full resistance	no	yes	yes	yes	no
Allows direct isolate-specific conclusion for combination therapy	no	no	no	yes ^2^	no	no	yes
Manual handling time	5–10 min	3–5 min	5–10 min, two sets	20–30 min	60 min	3 min	3 min
Time to result ^3^	1–4 h	12–18 h	15 min	18 h	30 h	1–30 min	15–60 min
Suitable as initial test	no	yes	no	yes	no	yes	yes
Suitable for automated performance	yes	yes	no	no	no	yes	yes
Approximate cost per test unit (EUR)	30	5–10	15 + 15 ^1^	2	2	4	4
Approximate cost for special equipment ^4^ (EUR)	12,000	40,000	0	0	0	0	0
Approximate cost for MALDI TOF MS for species identification. ^4^ (cost for device and cost for single ID, EUR)	not required	200,000 1-2/ID	200,000 1-2/ID	200,000 1-2/ID	not required for result	not required for result	not required for result

^1^ Costs for two sets for investigation of *Enterobacterales* and *A. baumannii* spp. with characterized species (e.g., by MALDI-TOF-MS), application of a single test set can be appropriate. ^2^ When inhibition zone diameter or single colonies are considered. ^3^ Starting from completed agar plate culture. ^4^ Example without costs for maintenance and/or service contract. * EUCAST species- or group-specific breakpoints. ** Depending on screening cutoffs.

## Data Availability

The data presented in this study are available on direct request from the author due to the very extensive data collection during the experiments.
